# Adenosine A_2A_ Receptor Blockade Modulates Glucocorticoid-Induced Morphological Alterations in Axons, But Not in Dendrites, of Hippocampal Neurons

**DOI:** 10.3389/fphar.2018.00219

**Published:** 2018-03-19

**Authors:** Helena Pinheiro, Rita Gaspar, Filipa I. Baptista, Carlos A. Fontes-Ribeiro, António F. Ambrósio, Catarina A. Gomes

**Affiliations:** ^1^Coimbra Institute for Clinical and Biomedical Research, Faculty of Medicine, University of Coimbra, Coimbra, Portugal; ^2^Center for Innovation in Biomedicine and Biotechnology, University of Coimbra, Coimbra, Portugal; ^3^Faculty of Medicine, University of Coimbra, Coimbra, Portugal

**Keywords:** dexamethasone, adenosine A_2A_ receptor, development, hippocampal neurons, morphology

## Abstract

The exposure to supra-physiological levels of glucocorticoids in prenatal life can lead to a long-term impact in brain cytoarchitecture, increasing the susceptibility to neuropsychiatric disorders. Dexamethasone, an exogenous glucocorticoid widely used in pregnant women in risk of preterm delivery, is associated with higher rates of neuropsychiatric conditions throughout life of the descendants. In animal models, prenatal dexamethasone exposure leads to anxious-like behavior and increased susceptibility to depressive-like behavior in adulthood, concomitant with alterations in neuronal morphology in brain regions implicated in the control of emotions and mood. The pharmacologic blockade of the purinergic adenosine A_2A_ receptor, which was previously described as anxiolytic, is also able to modulate neuronal morphology, namely in the hippocampus. Additionally, recent observations point to an interaction between glucocorticoid receptors (GRs) and adenosine A_2A_ receptors. In this work, we explored the impact of dexamethasone on neuronal morphology, and the putative implication of adenosine A_2A_ receptor in the mediation of dexamethasone effects. We report that *in vitro* hippocampal neurons exposed to dexamethasone (250 nM), in the early phases of development, exhibit a polarized morphology alteration: dendritic atrophy and axonal hypertrophy. While the effect of dexamethasone in the axon is dependent on the activation of adenosine A_2A_ receptor, the effect in the dendrites relies on the activation of GRs, regardless of the activation of adenosine A_2A_ receptor. These results support the hypothesis of the interaction between GRs and adenosine A_2A_ receptors and the potential therapeutic value of modulating adenosine A_2A_ receptors activation in order to prevent glucocorticoid-induced alterations in developing neurons.

## Introduction

The regulation of glucocorticoid (GC) levels during pregnancy is a major governing mechanism for the transition of the fetus to the extra-uterine life. During pregnancy, the levels of GC in the fetus are maintained lower than the mother’s circulating levels and, toward the delivery, the intrauterine levels of GC rise, inducing fetal maturation (Thorburn et al., 1977). Once in the circulation, GC exerts a plethora of effects at the peripheral level and in the brain, by binding to mineralocorticoid (MR) and glucocorticoid receptors (GRs). Since endogenous GC have a higher affinity to MR, low levels of GC bind preferentially these receptors (Myers et al., 2014). Under stress conditions, the fetal hypothalamic-pituitary-adrenal (HPA) axis is activated in the earlier stages of development, inducing tissue differentiation, with detrimental effects later in life (Fowden and Forhead, 2015).

The administration of GC during prenatal and early life development mimics early-stress effects, being highly concerning. However, synthetic GC, such as dexamethasone (DEX), administrated in women at risk of preterm delivery to accelerate fetal lung maturation, are a crucial clinical tool to increase preterm infants survival (Brownfoot et al., 2008). Nevertheless, synthetic GC are up to 20 times more potent than endogenous GC and have higher affinity to GR (contrasting with endogenous GC), triggering different mechanisms likely implicated in their detrimental effects (Fowden and Forhead, 2015). Indeed, the antenatal exposure to synthetic GC was shown, both in humans and animal models, to have long-term effects on HPA axis regulation (Nagano et al., 2008), brain structure and behavior, neurosensory, neuroendocrine, and cardio-metabolic functions (Constantinof et al., 2016).

A brief antenatal exposure to DEX induces long-term behavioral alterations, such as decreased locomotor activity and exploratory behavior, increased susceptibility to depressive-like behavior (Oliveira et al., 2006), anxious-like behavior (Caetano et al., 2016), and altered fear-response in adulthood (Oliveira et al., 2012). The antenatal exposure to GC affects the normal development of the hippocampus, leading to a decrease in hippocampus size and an increase in the number of apoptotic cells during early life (Noorlander et al., 2014). Alterations in the hippocampal structure were reported also in models of early life stress induced by maternal separation, such as atrophy of mossy fiber density (Huot et al., 2002) and dendrites (Batalha et al., 2013). Thus, cytoarchitecture alterations in neurons due to DEX exposure may underlay the behavioral alterations.

Recent observations of A_2A_ receptor (A_2A_R)-GR interaction in the hippocampus (Batalha et al., 2016) suggest that that A_2A_R may be modulating DEX-induced effects in hippocampal neuronal cytoarchitecture during development. Indeed, the modulation of A_2A_R has been regarded as a valuable therapeutic target in neuropsychiatric disorders (Cunha et al., 2008) and in the regulation of neuron morphology. The activation of A_2A_R in neuronal differentiated PC12 cells demonstrated that A_2A_R contributes to the increase in the number and length of neurites (Cheng et al., 2002; Charles et al., 2003). In primary cortical neurons, the activation of A_2A_R increases axonal elongation and dendritic branching during neuronal development (Ribeiro et al., 2016). The modulation of neuronal morphology by A_2A_R was also reported *in vivo.* Both the administration of caffeine in early life, a non-selective antagonist (Juárez-Méndez et al., 2006) and the treatment with a specific A_2A_R antagonist in adulthood (Batalha et al., 2013) lead to alterations in neurons morphology, demonstrating that the blockade of A_2A_R has an impact *in vivo* throughout all life span.

To test the hypothesis of A_2A_R-GR interaction in hippocampal neuronal morphology we analyzed the effects of exposure to DEX on the morphology of hippocampal neurons during early development in the presence and absence of an A_2A_R selective antagonist.

We report that DEX exposure induces a differential effect in the dendrites and axon of developing hippocampal neurons, characterized by dendritic atrophy and axonal hypertrophy. Whereas the effect in the increase in axonal length was dependent on the activation of A_2A_R, the effect in the dendrites depends on the activation of GR, and not on A_2A_R. These data suggest that the effects of DEX during development rely on distinct mechanisms in the different neuronal compartments.

## Materials and Methods

### Primary Rat Hippocampal Neuronal Cultures

Primary cultures of hippocampal neurons were obtained from Wistar rats, as previously described (Baptista et al., 2013). Pregnant females (gestational day 18) were anesthetized with isoflurane, and sacrificed by cervical dislocation. Pups were delivered by cesarean operation and sacrificed by decapitation using surgical scissors. Briefly, the hippocampi from each hemisphere were macrodissected and dissociated chemically in a 0.15% trypsin solution (Sigma-Aldrich). Trypsinization reaction was blocked with 10% fetal bovine serum. Then, the hippocampi were mechanically dissociated in Neurobasal medium (Gibco) 0.025 mM glutamate (supplemented with 0.5 mM L-glutamine (Sigma), 2% B27, 0.1% gentamycin (Gibco) and plated at a low density (3000 cells/coverslip) in 16 mm coverslips previously coated with poly-D-lysine (0.1 mg/ml, Sigma). Hippocampal neurons cultures were maintained in an incubator at 37°C, 5% CO_2_, until the end of the experiments. Four days after plating, at day *in vitro* (DIV) 4, half of the total medium volume was replaced by supplemented Neurobasal medium without glutamate, to avoid excitotoxicity.

All procedures involving animals were approved by the Animal Welfare Committee of the Faculty of Medicine of the University of Coimbra and were conducted in accordance with the European Community directive guidelines for the use of animals in laboratory (2010/63/EU), transposed into the Portuguese law in 2013 (Decreto-Lei 113/2013).

### Pharmacological Treatment

At DIV1, hippocampal neurons were treated with DEX (250 nM, Acros Organics), a concentration that leads to GR nuclear translocation under the control of adenosine A_2A_ receptors (unpublished data), and/or the selective A_2A_R antagonist SCH58261 (SCH, 50 nM, Tocris) [this concentration is selective for A_2A_R (Zocchi et al., 1996)], and/or the GR antagonist (RU486) mifepristone (MIF; 1 μM, Tocris) and the selective A_2A_R agonist CGS21680 (CGS, 30 nM). DEX binds preferentially to GR (Kornel et al., 1982) and this concentration of MIF is able to abolish DEX effects *in vitro* (Kamradt et al., 2000; Kimura et al., 2011). When the effects of DEX were tested in the presence of the A_2A_R antagonist, SCH was added 15 min before DEX, whereas in the case of the GR antagonist, MIF was added immediately before DEX.

### Immunocytochemistry

Hippocampal neurons were fixed in 4% PFA and 4% sucrose in PBS solution (137 mM NaCl, 2.7 mM KCl, 10 mM NaH_2_PO_4_.2H_2_O, 1.8 mM KH_2_PO_4_ in miliQ water, pH = 7.4) for 10 min, at RT. After permeabilization/blocking (PBS 5% BSA, NZYtech, 0.1% Triton X-100, Sigma), coverslips were incubated overnight with the primary antibody (1:1000, polyclonal rabbit anti-TUJ1, Covance), at 4°C, and for 2 h at RT with the secondary antibody (1:1000, polyclonal goat anti-rabbit, Thermo Fisher Scientific) after washing with PBS solution. Then, coverslips were incubated for 10 min with DAPI (1:5000 in PBS, Invitrogen) to stain nuclei, and mounted on microscope slides with glycergel mounting medium (DAKO).

### Morphometric Analysis of Hippocampal Neurons

Neuronal morphology was analyzed at DIV2 and DIV5, to evaluate the influence of the pharmacological treatments upon the initial development of the axon at DIV2 and the elongation of the dendrites at DIV5 (Dotti et al., 1988, #438). For naming purposes, in this study, the major processes at DIV2 were solely considered as axons. Images of neurons were acquired in a fluorescence microscope Zeiss Axio Imager 2 linked to Zeiss AxioCam, using a 20× objective lens (Plan Apochromat 20×/0.8) and processed by Zen Blue software (Zeiss). The settings of the acquisition were maintained throughout all experiments. Two main criteria were taken into consideration for the selection of neurons: the acquisition of neurons whose neurites were clearly distinguishable and not overlaid with others, and the proximity to other neurons (in a radius of 1000 μm) to avoid morphologic alterations due to lack of trophic support derived from other neurons.

Images were imported to the Neurolucida software (MBF Bioscience) and distinguished axons and dendrites were manually reconstructed, taking into consideration their morphological differences, by a researcher blinded to the treatment conditions. The major branch in each cell with a constant caliber was regarded as the axon, whereas the smaller neurites with taper ending were considered dendrites. All ramifications were considered, regardless of their length. At DIV2, 180 cells were reconstructed for each condition, in a total of six independent experiments. At DIV5, 120 cells were reconstructed for each condition, in a total of five independent experiments.

Morphometric data (branched structure analysis) was obtained in Neurolucida Explorer software, and the number of axons/dendrites, mean length of axons/dendrites, and total numbers of ramifications of each were analyzed.

### Statistical Analysis

Statistical analysis was carried out in GraphPad Prism version 5 (GraphPad Software Inc.). All graphic values are expressed as mean ± standard error of the mean (SEM). Comparison between two independent means was done by Student’s *t*-test. To assess differences between three groups, a one-way analysis of variance (ANOVA) was used, followed by a Tukey’s Multiple Comparison Test, to compare all groups. Differences were considered significant at *p* < 0.05.

## Results

### Exposure to DEX Has a Differential Effect in Different Neuronal Compartments, Inducing Axon Hypertrophy and Dendrite Atrophy

**FIGURE 1:**
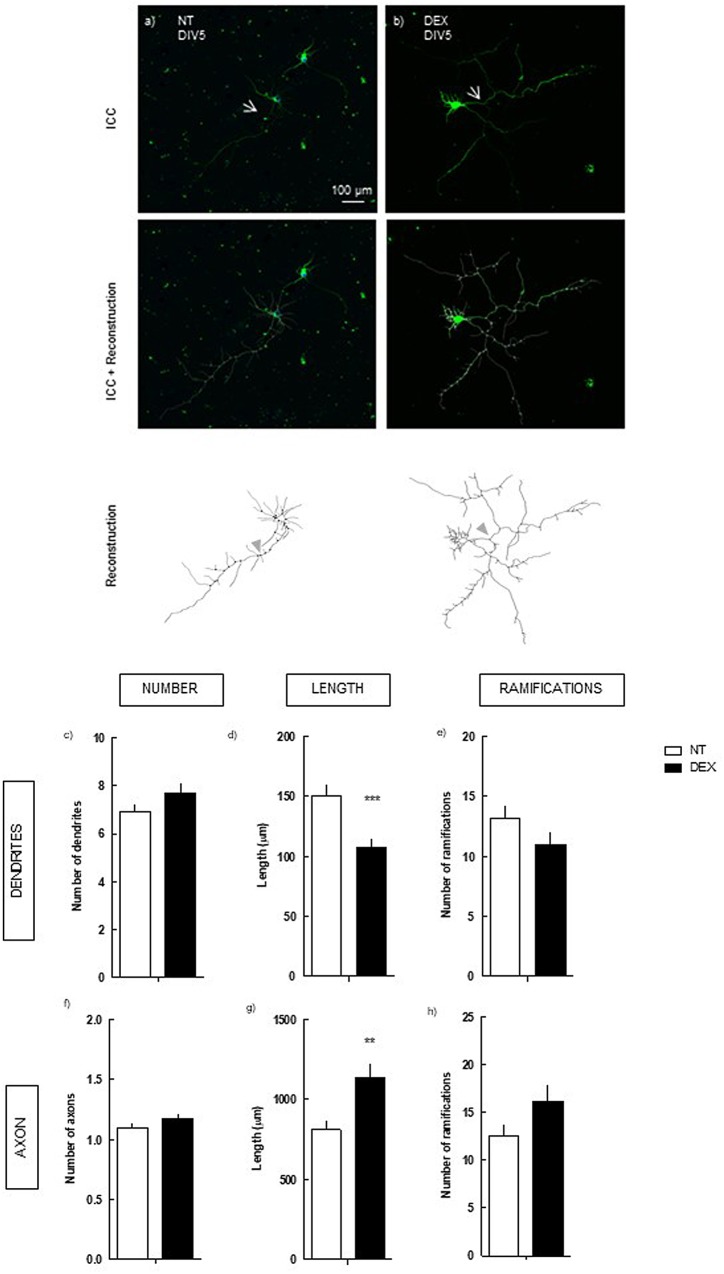
Effect of DEX treatment (4 days treatment) on hippocampal neurons (DIV5). Hippocampal neurons from ED18 rats were cultured *in vitro* for 5 days (DIV5) and treated with DEX (250 nM) at 24 h in culture. Neuronal morphology was assessed by manual reconstruction in Neurolucida software **(a,b)** and morphometric data were acquired in Neurolucida Explorer, regarding the number **(c)**, length **(d)** and number of ramifications **(e)** of dendrites and the number **(f)**, length **(g)**, and number of ramifications **(h)** of axons (identified with an arrow in the representative images). Results are expressed as mean ± SEM of 120 cells, from five independent experiments. Statistical significance was assessed by *t*-student test: ^∗∗^*p* < 0.01, ^∗∗∗^*p* < 0.001, comparing DEX treatment with NT. ICC, immunocytochemistry; NT, non-treated; DEX, dexamethasone.

To assess the effects of DEX upon neuronal morphogenesis, primary hippocampal neurons were treated with DEX (250 nM) after 24 h in culture and neuronal morphology was analyzed after 2 and 5 days in culture (**Figure 1**), by manual reconstruction, using Neurolucida software. Morphometric data were analyzed considering the number and length of dendrites and axons, and the respective ramification.

We observed that DEX treatment did not alter neuronal morphology after 2 days in culture (Supplementary Figure 1). Contrastingly, at 5 days in culture DEX exposure induced a pronounced decrease in the mean length of the dendrites: 107.6 ± 6.6 μm (*p* < 0.001), as compared with non-treated (NT; 150.7 ± 8.1 μm) (**Figure 1d**). However, no statistical effect was detected upon their number and number of ramifications. In the axon, DEX induced the opposite effect, increasing its length: 1139.2 ± 86.1 μm (*p* < 0.01), as compared with NT (811.5 ± 57.6 μm) (**Figure 1g**).

Thus, exposure of hippocampal neurons to DEX induces a contrasting modulation of neuronal morphology, characterized by axonal hypertrophy and dendritic atrophy.

### DEX-Induced Increase in Axon Length Is Dependent on the Activation of Adenosine A_2A_ Receptors

**FIGURE 2:**
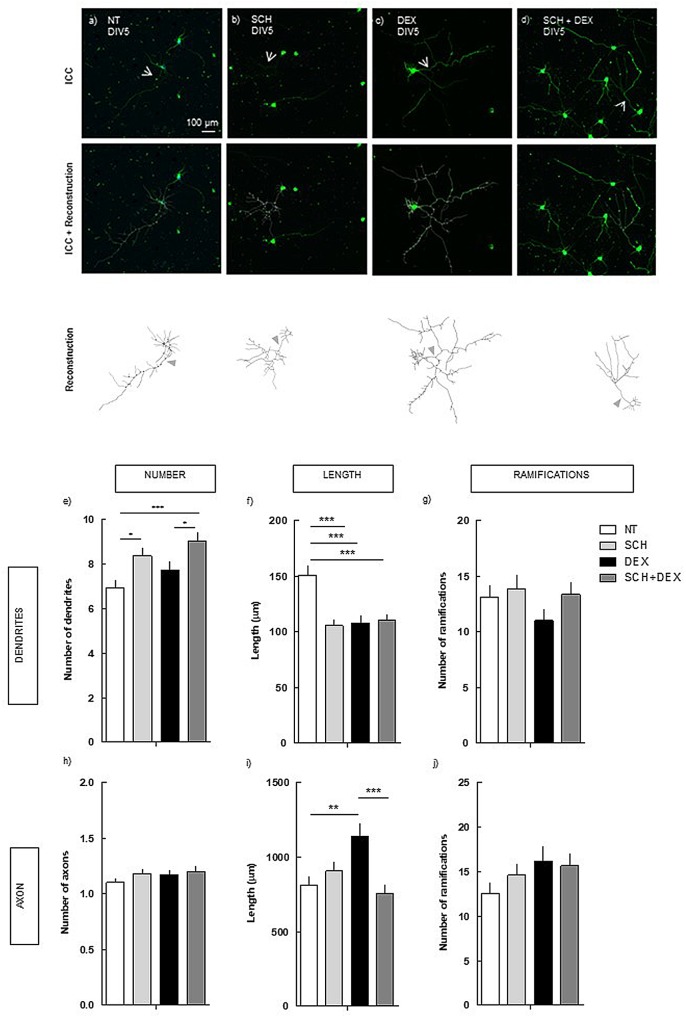
Effect of the blockade of A_2A_R *per se* and on DEX exposure (4 days treatment) in hippocampal neurons (DIV5). Hippocampal neurons from ED18 rats were cultured *in vitro* for 5 days (DIV5). Cultured neurons were treated with the A_2A_R antagonist, SCH (50 nM), or with the A_2A_R antagonist and/or DEX (250 nM; at 24 h in culture). SCH was added 15 min before DEX. Neuronal morphology was assessed by manual reconstruction in Neurolucida software **(a–d)** and morphometric data were acquired in Neurolucida Explorer, regarding the number **(e)**, length **(f)**, and number of ramifications **(g)** of dendrites and the number **(h)**, length **(i)**, and number of ramifications **(j)** of axons (identified with an arrow in the representative images). Results are expressed as mean ± SEM of 120 cells, from five independent experiments. Statistical significance was assessed by one-way ANOVA followed by Tukey’s Multiple Comparison Test: ^∗^*p* < 0.05, ^∗∗^*p* < 0.01, ^∗∗∗^*p* < 0.001, as indicated by the horizontal lines above the columns. ICC, immunocytochemistry; NT, non-treated; SCH, SCH58261, A_2A_R antagonist; DEX, dexamethasone; SCH+DEX, SCH58261 + Dexamethasone.

To understand if the activation of A_2A_R is implicated in the modulation of neuronal morphology induced by DEX, we analyzed the impact of DEX in hippocampal neurons under the pharmacological blockade of A_2A_R, using a selective antagonist (SCH58261) (**Figure 2**). Hence, primary hippocampal neurons were treated with 250 nM DEX in the absence or presence of 50 nM SCH58261, and neuronal morphology was analyzed after 5 days in culture.

The blockade of A_2A_R was not able to prevent the atrophy in the length of the dendrites induced by DEX (110.0 ± 5.4 μm), comparing with DEX alone (107.6 ± 6.6 μm). Indeed, the effect of A_2A_R plus DEX, which induced a similar decrease as DEX alone, was significantly different (*p* < 0.001) from NT cells (150.7 ± 8.1 μm). The treatment with SCH *per se* induced a decrease in the length of the dendrites similar to the effect of DEX treatment (**Figure 2f**), indicating that both DEX and SCH induce similar alterations in dendrites’ length.

Regarding the number of dendrites, although DEX treatment did not induce a significant alteration, there was a tendency to increase. Regarding the treatment with SCH, there was an increase in the number of dendrites, both in the absence (8.4 ± 0.3; *p* < 0.05) and presence of DEX (9.0 ± 0.4; *p* < 0.001), comparing with NT (6.9 ± 0.3).

Conversely, in the axon, A_2A_R blockade prevented the increase (*p* < 0.001) in the length induced by DEX (752.4 ± 60.8 μm), comparing with DEX alone (1139.2 ± 86.1 μm; **Figure 2i**), having a similar length as in NT cells (811.5 ± 57.6 μm; n.s.).

These findings show that there is an uncouple in the mechanisms underlying the effect of DEX exposure in dendrites and axon, demonstrating the requirement of the activation of different receptors.

### DEX-Mediated Increase in Axon Length Is Not Exclusively Modulated by the Activation of A_2A_R

Given that the blockade of A_2A_R *per se* led to alterations in neuronal morphology similar to those observed in the blockade of A_2A_R combined with DEX, we aimed to clarify if the rescue of the axon length is not mediated solely by the blockade of A_2A_R activation by tonic adenosine, instead of a modulation dependent on GR. Thus, we analyzed the effect of a selective A_2A_R agonist, 30 nM CGS 21680, in neuronal morphology.

**FIGURE 3:**
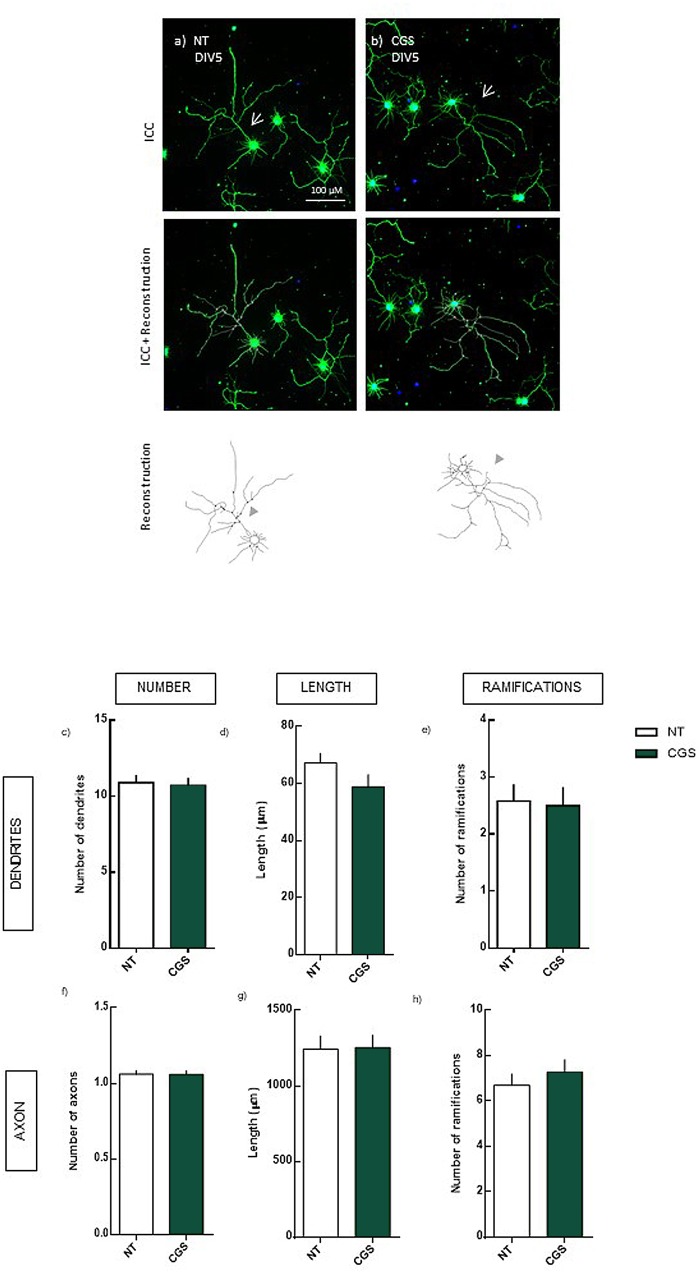
Effect of the selective activation of A_2A_R (4 days treatment) in hippocampal neurons (DIV5). Hippocampal neurons from ED18 rats were cultured *in vitro* for 5 days (DIV5). Cultured neurons were treated with the A_2A_R agonist, CGS (30 nM), at 24 h in culture. Neuronal morphology was assessed by manual reconstruction in Neurolucida software **(a,b)** and morphometric data were acquired in Neurolucida Explorer, regarding the number **(c)**, length **(d)**, and number of ramifications **(e)** of dendrites and the number **(f)**, length **(g)**, and number of ramifications **(h)** of axons (identified with an arrow in the representative images). Results are expressed as mean ± SEM of 97–100 cells, from four independent experiments. No statistical significance comparing NT with CGS treatment, assessed by *t*-student test. ICC, immunocytochemistry; NT, non-treated; CGS, CGS21680, A_2A_R agonist.

We observed that, in the absence of DEX, the activation of A_2A_R does not lead to significant alterations in neuronal morphology, not in the dendrites or axon (**Figure 3**).

These results indicate that the DEX-induced axonal hypertrophy, although dependent on the activation of A_2A_R requires also the activation of GR, indicating a crosstalk between A_2A_R/GR.

Surprisingly, even though the blockade of A_2A_R *per se* led to alterations in dendrites’ morphology, these alterations were not observed by the selective activation of A_2A_R. This indicates that the maintenance of adenosine tonic levels is crucial for neuronal morphology, although the overactivation of A_2A_R does not alter morphology.

### DEX-Induced Decrease in Dendrite Length Is Dependent on the Activation of Glucocorticoids Receptors

**FIGURE 4:**
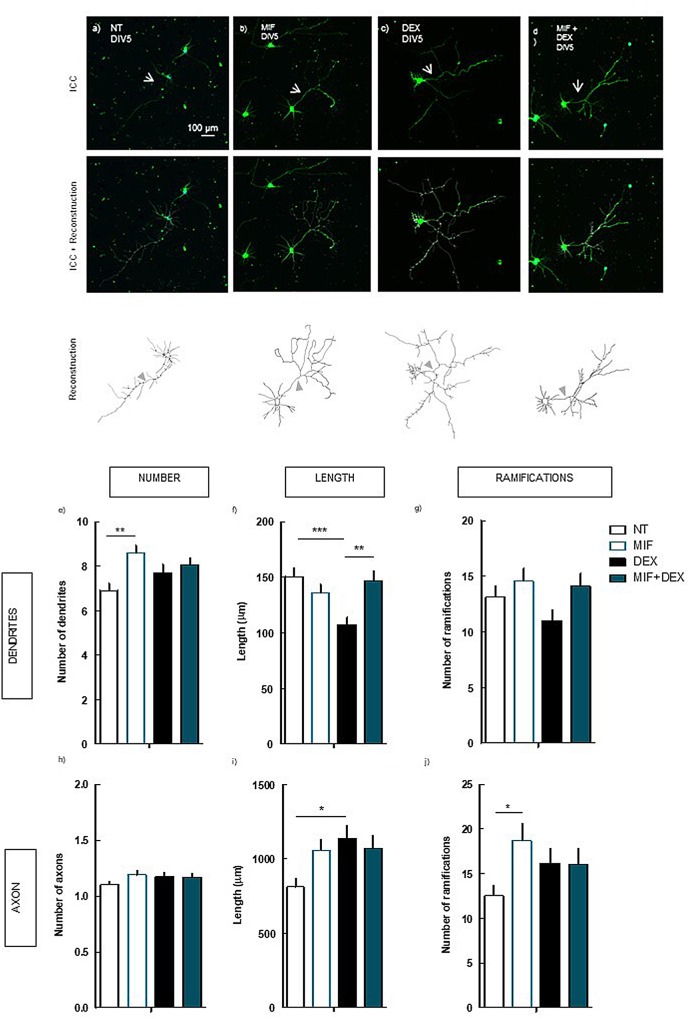
Effect of the blockade of GR *per se* and on DEX exposure (4 days treatment) in hippocampal neurons (DIV5). Hippocampal neurons from ED18 rats were cultured *in vitro* for 5 days (DIV5). Cultured neurons were treated with the GR antagonist, MIF (1 μM), or with the GR antagonist and/or DEX (250 nM; at 24 h in culture). Neuronal morphology was assessed by manual reconstruction in Neurolucida software **(a–d)** and morphometric data were acquired in Neurolucida Explorer, regarding the number **(e)**, length **(f)**, and number of ramifications **(g)** of dendrites and the number **(h)**, length **(i)**, and number of ramifications **(j)** of axons (identified with an arrow in the representative images). Results are expressed as mean ± SEM of 120 cells, from five independent experiments. Statistical significance was assessed by one-way ANOVA followed by Tukey’s Multiple Comparison Test: ^∗^*p* < 0.05, ^∗∗^*p* < 0.01, ^∗∗∗^*p* < 0.001, as indicated by the horizontal lines above the columns. ICC, immunocytochemistry; NT, non-treated; MIF, mifepristone, GR antagonist.; DEX, dexamethasone; MIF+DEX, mifepristone + dexamethasone.

Considering that synthetic glucocorticoids, namely DEX, have a high affinity to GR (Kornel et al., 1982), we sought to confirm if the effects of DEX on the morphology of neurons are dependent on the activation of GR. To test this hypothesis, primary hippocampal neurons were treated with 250 nM DEX, in the absence or presence of the antagonist of GR, 1 μM MIF (**Figure 4**).

The blockade of GR prevented the alteration in the length of the dendrites induced by DEX (147.1 ± 8.6 μm; *p* < 0.001) as compared with DEX alone (107.6 ± 6.6 μm) (**Figure 4f**), to values similar to NT cells (150.7 ± 8.1 μm; n.s.). Additionally, the treatment with MIF *per se* led to an increase (*p* < 0.01) in the number of dendrites (8.6 ± 0.4) as compared with NT (6.9 ± 0.3) (**Figure 4e**), demonstrating a hypertrophic effect of the blockade of endogenous glucocorticoids.

The increase in the length of the axon induced by DEX was not prevented by the blockade of GR (1069.1 ± 88.7 μm), as compared with DEX alone (1139.2 ± 86.1 μm, n.s.) (**Figure 4i**). In contrast to the observations in dendrites, the effect of DEX in the presence of MIF in the axon is similar to the effect of DEX alone, indicative of two different mechanisms overriding DEX effects in axon and dendrites.

Indeed, we demonstrate that whereas the effect of DEX in the dendritic morphology depends on the direct activation of GR, the effects on the axon are modulated by the activation of A_2A_R.

The observation that the blockade of GR *per se* leads to an increase (*p* < 0.05) in the number of ramifications in the axon (18.7 ± 2.0) as compared with NT (12.5 ± 1.2), is in line with the possible hypertrophic effect of blocking the action of endogenous glucocorticoids raised above. However, the hypertrophic effect of GR blockade could be also due to an increase in the activation of mineralocorticoid receptors by tonic glucocorticoids, rather than the lack of GR activation.

## Discussion

The development of the brain is tightly regulated by environmental factors. Thus, negative environmental stimuli, such as prenatal and early life stress, which lead to increased levels of glucocorticoids can have a long-term impact in the brain cytoarchitecture and function (Oliveira et al., 2006, 2012; Leão et al., 2007). Similarly, glucocorticoid treatments, such as DEX, also lead to detrimental effects in the brain. However, the implementation of these approaches in women in risk of preterm birth was an undoubtable advance in the increase of survival rates of premature newborns (Brownfoot et al., 2008). Therefore, it is crucial to understand the modulating effects of glucocorticoid exposure in the developing brain in order to develop new pharmacological approaches to circumvent their negative effects.

In the present work, we reported that a long-term exposure to DEX in developing hippocampal neurons leads to a differential modulation of neuronal morphology, characterized by dendrites’ atrophy and axonal hypertrophy. The observation that DEX did not alter neuronal morphology after 24 h of exposure (Supplementary Figure 1) indicates that DEX-mediated effects in morphology are delayed or restricted to later stages of development.

The delay in DEX effects is in line with previous observations in PC12 cells, in which the treatment with a low dose of DEX leads to an increase in cell growth only after 72 h of exposure, as indicated by the total protein/DNA ratio (Jameson et al., 2006). As that method does not discriminate cell morphology, the results do not oppose our observations, once the differential effect in dendrites and axon may lead to an overall increase in cell area. However, in a different study exploring the effect of high doses of DEX (5 μM) it was observed that DEX exposure for 48 h leads to an overall inhibition of neurite development (Beaujean et al., 2003), contrasting with the present observations that DEX does not induce any effect 24 h after treatment. This discrepancy could be due to the higher concentration of DEX or differences in the susceptibility of the PC12 cell line comparing with cultured hippocampal neurons. In a different cell line, HiB5 cells, it was also observed that the exposure to DEX in a concentration in the same range of the concentration in the present study (10^-7^ M) also inhibits neurite development (Son et al., 2001), which may indicate that cell lines and hippocampal primary neurons may respond differently to DEX exposure.

Overall, there is a lack of understanding regarding the direct effect of DEX in neurons. Over the last two decades, few studies were developed to explore the effects of DEX exposure *in vitro*, which could highly contribute to the dissection of the effects observed at the organism level.

In *in vivo* models, it is interesting to notice that several reports showed dendritic atrophy, resulting from either stress or glucocorticoid exposure in brain regions such as the prefrontal cortex (Brown et al., 2005; Anderson et al., 2016) and the hippocampus (Sousa et al., 1999, 2000; Silva-Gómez et al., 2013). However, alterations in axonal morphology were not yet reported, probably due to the difficulties associated with the analysis of axonal morphology *in vivo* given the higher complexity of this cellular compartment. Nevertheless, alterations in axonal morphology can alter brain connectivity and are implicated in psychiatric disorders. In a genetic model of schizophrenia, it was observed an impair in axonal growth and branching, which leads to cognitive deficits and high incidence of emotional problems (Mukai et al., 2015).

Although the activation of A_2A_R in the brain is well documented as a modulator of synaptic transmission and plasticity, these receptors are also implicated in neuronal morphologic development *in vivo*. The modulation of A_2A_R activation was previously reported to impair brain connectivity trough axonal development alterations in a model of *in utero* exposure to an A_2A_R antagonist. This impairment leads to a delay in axonal migration, which is associated to cognitive deficits in adulthood (Silva et al., 2013). Considering the report that A_2A_R activation induces axonal elongation *in vitro* by inducing an increase in microtubule dynamics and growth speed (Ribeiro et al., 2016), we speculate that the modulation of DEX axonal effect by A_2A_R could be due to a similar mechanism.

However, in the present study, the treatment with an A_2A_R agonist did not alter neuronal morphology, suggesting that the effects of A_2A_R modulation in hippocampal neurons are different from the ones in cortical neurons, or that the differences in these results are due to the analysis of neuronal morphology in a different time interval in neuronal development, once we analyzed morphology at day 5 in culture whereas in the referred study the analysis of cortical neurons was at day 3. Indeed, it is interesting that although the activation of A_2A_R is necessary for the effects of DEX in the axon, as seen by the blockade of DEX-effect in the presence of the A_2A_R antagonist, the activation of A_2A_R does not alter neuronal morphology. This indicates that DEX-effect is not exclusively mediated by A_2A_R, further supporting the hypothesis of a GR–A_2A_R crosstalk.

Interestingly, in microglia, we previously described such a putative interaction. It is well documented that A_2A_R are also important regulators of microglia morphology (Gyoneva et al., 2009, 2014; Orr et al., 2009; Caetano et al., 2016). We described that *in utero* exposure to DEX induces long-term alterations in microglia morphology which correlate with anxiety-like behavior, and that this alterations are recovered by A_2A_R blockade in a gender-specific manner (Caetano et al., 2016). However, we observed only a partial recovery of microglia morphologic alterations induced by DEX accompanied by a complete recover in behavior might indicate additional targets mediating A_2A_R blockade effects, namely neurons. This prompted us to understand the direct effects of DEX exposure in neurons, and the therapeutic potential of A_2A_R blockade.

Although we previously observed a DEX and A_2A_R gender-specific effect in microglia *in vivo*, in this study, we did not discriminate the sex of the fetuses, due to restrictions in the numbers of animals available to perform neuronal cultures. However, since the neurons suffer a reprogramming upon culture, and are from then on cultured in the same conditions, we do not expect such striking differences in the data obtained.

In what concerns GR–A_2A_R crosstalk, it was recently described that the blockade of A_2A_R activation blocks GR translocation to the nucleus, thus impairing GR activation-induced transcriptional alterations (Batalha et al., 2016). Accordingly, the effects of DEX in the axon are blocked by the simultaneous treatment with the A_2A_R antagonist, suggesting that the axon hypertrophy is dependent on GR transcriptional activity.

However, there is a discrepancy between DEX-effects on dendrites and axons. The dendritic atrophy is independent of the blockade of A_2A_R, which might suggest that the effects on dendrites may be due to GR effects that do not require nuclear translocation and are due to GR non-genomic effects.

On the other hand, the axonal effects were abolished by the blockade of A_2A_R, which disrupts nuclear translocation, indicating that DEX effect on the axon depends on GR genomic action. Although, it is interesting to notice that the DEX effect was not present after 24 h of DEX exposure in either the dendrites or the axon. If the effects in the dendrites are indeed modulated by GR non-genoimc effects, the decrease in dendrites length could potentially be achieved earlier than the increase in the axon, once it would not depend on transcriptional alterations. This point could be clarified by a closer monitoring of the morphological alterations induced by DEX to pinpoint a more accurate moment of the outset of dendritic and axonal alterations.

Additionally, according to this hypothesis, the effect of DEX in the axon should be similarly blocked in the presence of GR antagonist or A_2A_R antagonist, once both should block the receptor translocation to the nucleus. These results would be explained if the increase of GR nuclear translocation upon activation of A_2A_R was independent on the presence of GR ligands, as DEX.

It is also possible that the distribution of A_2A_R is differential in axon and dendrites, leading to a significant effect upon the GR signaling in the axon, but not in the dendrites. It is important to address all these hypotheses to further understand the nature of GR–A_2A_R interaction and, consequently, the putative pharmacological modulation of A_2A_R activation.

Furthermore, since the present results indicate that DEX effects on axonal morphology are dependent on the activation of A_2A_R, clarifying the mechanisms underlying these receptors crosstalk can be crucial to understand DEX action on neuronal morphology. As it was previously described, the activation of A_2A_R in cortical neurons promotes the growth speed of microtubules in the axonal growth cone, leading to axonal elongation (Ribeiro et al., 2016). In future work, it is also important to understand how glucocorticoid exposure modulates neuronal morphology, considering the possibility that the effect of DEX may be as well dependent on microtubule dynamics.

Understanding the mechanistic relation of GR and A_2A_R and the nature of A_2A_R morphological modulation in neurons and other cell types is crucial to the further development of A_2A_R blockade therapies.

The genetic and pharmacological blockade of A_2A_R was described as anxiolytic in a model of chronic stress in males, both as a preventive and therapeutic tool (Kaster et al., 2015). Additionally, the chronic blockade of A_2A_R in adulthood is able to revert behavioral, electrophysiological and neuronal morphological alterations induced by maternal separation (Batalha et al., 2013), clearly demonstrating its pharmacological potential in psychiatric pathologies.

However, the response can differ according to gender (Caetano et al., 2016) and it can be prejudicial when administered during development. The blockade of A_2A_R during development leads to a delay in axonal migration and consequent neuronal excitability, resulting in an increase in the susceptibility to seizures (Silva et al., 2013). Thus, understanding the different pathways induced by A_2A_R activation in these conditions is essential to further pursue the neuropsychiatric pharmacological advantages of its blockade.

## Conclusion

Although the use of synthetic GC in clinics is essential due to their beneficial effects in several pathologies, it is important to have a full understanding of their potential deleterious effects, namely in the CNS. The previous described effects of GC exposure in the morphology and physiology of neurons probably underlie the observed neuropsychiatric disturbances. Thus, given the therapeutic potential of A_2A_R blockade in neuropsychiatric disorders, it is essential to fully comprehend the mechanisms of GR and A_2A_R interaction, as well as the mechanisms responsible for the modulation of the brain cytoarchitecture by these receptors.

## Author Contributions

HP designed the experiments with CG, performed the experiments, and wrote the manuscript. RG performed hippocampal neurons primary cultures and immunocytochemistry for the experiment with A_2A_R agonist treatment. FB assisted in hippocampal neurons primary cultures and revised the manuscript. AA revised the manuscript. CG supervised HP, contributed to the design of the experiments, and revised the manuscript. All authors discussed the results, contributed to and approved the final manuscript.

## Conflict of Interest Statement

The authors declare that the research was conducted in the absence of any commercial or financial relationships that could be construed as a potential conflict of interest.
