# Supplementary figures and images for: Adenosine A2A Receptor Blockade Modulates Glucocorticoid-Induced Morphological Alterations in Axons, But Not in Dendrites, of Hippocampal Neurons
Source: Front Pharmacol. 2018 Mar 19;9:219. doi: 10.3389/fphar.2018.00219 (PMC5868516; doi:10.3389/fphar.2018.00219)

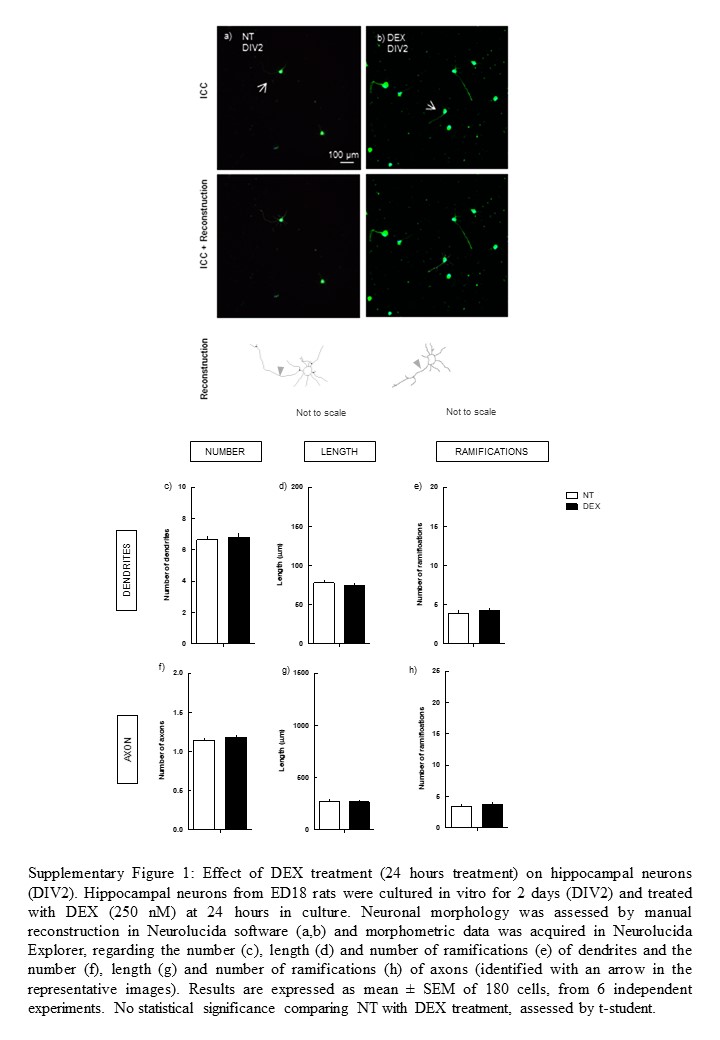

Supplement: Supplementary file 1 [file Image_1.JPEG]
